# Understanding COVID-19: From Origin to Potential Therapeutics

**DOI:** 10.3390/ijerph17165904

**Published:** 2020-08-14

**Authors:** Muhammad Moazzam, Muhammad Imran Sajid, Hamza Shahid, Jahanzaib Butt, Irfan Bashir, Muhammad Jamshaid, Amir Nasrolahi Shirazi, Rakesh Kumar Tiwari

**Affiliations:** 1 Faculty of Pharmacy, University of Central Punjab, Lahore 54700, Pakistan; moazzamshakeel91@gmail.com (M.M.); sajid@chapman.edu (M.I.S.); hamza.hashmi@ucp.edu.pk (H.S.); jahanzaib444@ucp.edu.pk (J.B.); irfan.bashir@ucp.edu.pk (I.B.); dr.jamshaid@ucp.edu.pk (M.J.); 2 Center for Targeted Drug Delivery, Department of Biomedical and Pharmaceutical Sciences, Chapman University School of Pharmacy, Harry and Diane Rinker Health Science Campus, Irvine, CA 92618, USA; 3 Foundation for Young Researchers, Lahore 54700, Pakistan; 4 Department of Pharmaceutical Sciences, College of Pharmacy, Marshall B. Ketchum University, Fullerton, CA 92831, USA; ashirazi@ketchum.edu

**Keywords:** COVID-19, cytokine storm, clinical features, dexamethasone, epidemiology, life cycle, pathogenesis, RT-PCR, remdesivir, SARS-CoV-2, therapeutics, vaccines

## Abstract

Currently, a global pandemic era of public health concerns is going on with the Coronavirus Disease 2019 (COVID-19), which is caused by severe acute respiratory syndrome coronavirus-2 (SARS-CoV-2). The first case of COVID-19 was reported from Wuhan’s Huanan seafood market in China late December 2019. Bats, pangolins, and snakes have been nominated as salient carriers of the virus. Thanks to its high pathogenicity, it can cause severe respiratory infections. Fever, dry cough, sore throat, pneumonia, septic shock, and ground-glass opacities are the foremost clinical manifestations of COVID-19. Immunocompromised patients are at high risk for COVID-19 infection and may lead to death. Scientist and government agencies around the globe are putting forward their best efforts and resources for the effective treatment of human coronavirus infections; however, neither vaccines nor antiviral drugs are available for the treatment of human coronaviruses (HCoV) infections such as SARS (severe acute respiratory syndrome), MERS (Middle Eastern respiratory syndrome), and COVID-19. Since the outbreak, a plethora of research and review articles have been published. Moreover, the mass media has bombarded the public with conflicting opinions about the pandemic. There is a dire need for accurate and reliable information concerning this pandemic. In this review, we have compiled the up to date information about the origins, evolution, epidemiology, and pathogenesis of this disease. Moreover, very few reports have addressed the clinical features and current status of treatment for COVID-19; we have adequately addressed these topics in detail in this review. Finally, a detailed account of clinical trials of vaccines and other therapeutics currently in progress has been delineated.

## 1. Introduction

The recent pandemic has affected more than seventeen million people across 215 countries and territories and caused deaths of more than 751.399 people as per reported data from John Hopkins University Coronavirus Resource Center on August 13, 2020. World Health Organization declared this pandemic a Public Health Emergency of International Concern (PHEIC) on 31 January 2020 [1]. The novel coronavirus responsible for this pandemic was named “2019-nCoV” and was later changed to “SARS-CoV-2” owing to its high similarity to severe acute respiratory syndrome coronavirus (SARS-CoV) [2]. Finally, it became popular as Coronavirus Disease 2019 (COVID-19) [3]. Scientists and governments around the globe are putting forward their best efforts and resources for the effective treatment of human coronavirus (HCoV) infections. However, neither vaccines nor antiviral drugs are approved for the treatment of HCoV infections such as SARS, MERS (Middle Eastern respiratory syndrome), and the COVID-19 [4,5,6,7]. The word “corona” is derived from Latin, meaning crown, as it possesses crown-like structure, hence called coronavirus. Coronaviruses possess a small size of approximately 65–125 nm in diameter and hold the nucleic material as single-stranded RNA. The similarity of the SARS-CoV-2 genome with SARS-CoV is about 79% [8]. SARS-CoV-2 is a type of beta coronavirus [9], belonging to the Coronavirinae subfamily and Coronaviridae family in order Nidovirales [10]. Alpha-, beta-, gamma-, and delta-coronaviruses are the subgroups of the coronavirus family. The alpha- and beta-coronaviruses originated from mammals, while gamma- and delta-coronaviruses originated from birds and pigs [11].

SARS-CoV and MERS-CoV originated from China in 2003 [12] and Saudi Arabia [13] in 2012, respectively, while the outbreak of COVID-19 began in December 2019 in Wuhan, China. Coronaviruses (CoVs) have shown significant pathogenicity against humans and other vertebrates [14]. These coronaviruses are highly contagious [15] and can cause severe respiratory infection [16] and acute respiratory distress syndrome (ARDS), followed by acute lung injury (ALI), leading to pulmonary failure and, ultimately, death [17]. The current strain of COVID-19 is less deadly than the previous MERS-CoV infection, with a mortality rate of 40%, and SARS-CoV, with a mortality rate of 10% [18,19]. Epidemiological investigations conducted by the World Health Organization (WHO) confirmed that the COVID-19 triggered by the Huanan South China seafood marketplace in Wuhan. However, no substantial evidence to exhibit that animal connection has been identified [20,21].

Further studies concluded that the genome sequence of COVID-19 is highly related to CoV infecting bats [22]. Recently, it has been shown that bat CoVs can infect humans without an intermediate carrier [23,24]. It is suggested that SARS-CoV-2 originated from the Chinese chrysanthemum bat as their genomic sequence is very much similar to SARS-CoV-2. Furthermore, there may be an intermediate host between humans and bats. The pangolin, an animal used by the Chinese for its meat and having specific medicinal value, is thought to be the source of this virus. Epidemiologists speculate that someone who purchased a pangolin from one of the wet markets in Wuhan got infected after consumption, setting forward the chain of transmission [25].

A plethora of research and review papers have been published since the onset of this pandemic [7]. Moreover, the mass media is bombarding the public with conflicting information that is often misleading and confusing. There is an urgent need for accurate and reliable information related to COVID-19. In this review, we have tried our best to compile up to date and accurate information, citing reliable and reputed journals, that will be helpful for the scientific community as well as the researchers working in this field. First, we thoroughly explained the origins and evolution of SARS-CoV-2, followed by a detailed account of its epidemiology and pathogenesis. There are very few reports that address the comprehensive clinical features of the disease. Furthermore, information about treatment options for COVID-19 is not adequate.

In this report, we have tried to gather the most updated and accurate data concerning this disease. In addition, information about all ongoing clinical trials of vaccines and other potential therapeutics has been addressed comprehensively. This review covers multiples aspects of COVID-19, including the structure of the virus, evolution process of the disease, epidemiologic outcomes, pathogenetic data, clinical features, diagnosis tools, treatment strategies, and prevention and control pathways.

## 2. Structure of SARS-CoV-2

**Figure 1:**
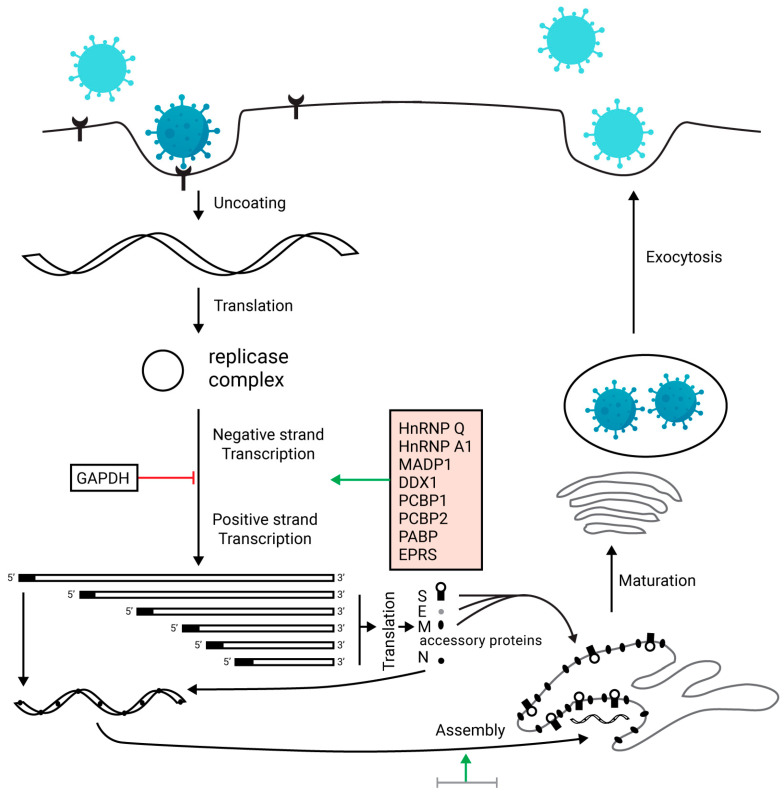
The life cycle of a coronavirus, modified, and used from Zhang et al. [27] with an open-access journal license. Upon entry to the host lungs, the CoV attaches to angiotensin-converting enzyme (ACE-2) receptors and releases its nuclear content into the cytoplasm. Translation of the RNA makes replicase complex, which results in the formation of transcripts of several proteins (named in pink box). Translation of these transcripts makes spike (S), envelope (E), membrane (M), nucleocapsid (N), and other accessory proteins. Assembly of these proteins produces a new virion that is matured and is released out of the cell to infect other cells.

The viral genome of SARS-CoV-2 is composed of 29,903 nucleotides and highly resembles SARS-like CoVs infecting bats with a nucleotide similarity of 89.1%. SARS-CoV-2, like other CoVs, possesses a large RNA genome and exhibits a unique replication strategy. Specifically, the virus has a nonsegmented positive-sense RNA genome with a 5′cap structure along with a 3′ poly (A) tail. This unique structure allows the replicase polyproteins to read the genome for translation. Two-thirds of the genome encodes nonstructural proteins (NSPs), while the remaining one-third encodes for structural and accessory proteins. Vital structural proteins are spike (S), membrane (M), envelope (E), and nucleocapsid (N) proteins. In addition, two viral replicase polyproteins termed PP1a and PP1ab are produced by the virus, which are modified into 16 mature NSPs [26,27]. Figure 1 illustrates the life cycle of the CoVs.

## 3. Evolution of COVID-19

It has been suggested that most of the coronavirus species infecting humans are found in the bat reservoir [28]. Unsurprisingly, several scientist groups have certified the genetic resemblance between SARS-CoV-2 and coronaviruses found in bats. A recent study showed that the gene sequence of novel coronavirus has 96.2% similarity to the coronaviruses found in bat (RaTG13), 89.1% similarity to SARS-CoV [26], and about 50% similarity to MERS-CoV [29,30]. It has been documented that both SARS-CoV-2 and SARS-CoV attach to the same receptor at the host named angiotensin-converting enzyme (ACE2). After analyzing and evaluating 103 genomes of SARS-CoV-2 by single nucleotide polymorphisms (SNPs), a recent study showed that the virus evolved into two L and S types. SNPs showed absolute linkage across the strain of SARS-CoV-2. Examination of these types revealed that the L-type (70%) is predominant as compared with the S-type (30%). The evolutionary inspection suggested that the S-type is the most ancient form of SARS-CoV-2. Several tests and analyses also showed that the L-type is more vigorous and cruel as compared with the S-type [31].

An unimaginably high degree of transmission raised a question of whether evolution was stimulated by mutations. Genetic mutation analysis is essential for understanding evolution [32]. A study was conducted to understand and evaluate the genetic mutations pattern, in which eighty-six genomes of SARS-CoV-2 were selected. All strains were identified in patients from Australia, China, France, England, Germany, Taiwan, Japan, Belgium, USA, and Vietnam. China was used as a reference to evaluate the sequence of the genome. SARS-CoV-2 presents a specific type of protein, a long ORF1ab polyprotein at the 5′ end with additional structural proteins, including envelope protein, spike glycoprotein, matrix, and nucleocapsid protein. The genetic examination identified three deletions in the genomes collected from Australia, the USA, and Japan. Out of three deletions, two deletions were noted in the ORF1ab polyprotein, and one deletion was noted in the 3′ end of the genome. Furthermore, changing antigenicity may be driven by the mutations that occurred in the spike glycoproteins, which may be the reason for the vigorous pathogenicity of SARS-CoV-2 [33].

Two precarious mutations were observed in the bat coronaviruses considered highly transmissible so far and are attributed to the current COVID-19 pandemic. Out of the two, the first improved the structure of spike proteins that appeared as projections on the outer surface of the virus. These modified spike proteins allow the virus to attach to the ACE2 receptor, which spans the respiratory tract lining. The second critical mutation permitted the virus to develop a protein dagger named furin, which can slice other proteins to cause the virus to bind firmly to the lung and throat cells. It is presumed that furin protein is the reason that COVID-19 is so deadly and contagious to humans [34]. Those mutations could have appeared while the virus was moving in bats. Another possibility of mutation is that it could have occurred in a person who was infected by the former type of virus but showed no sign and symptoms [35].

## 4. Epidemiology

As described earlier, SARS-CoV-2 originated from Wuhan, China, and has affected more than 20 million people across 212 countries and territories [14]. The transmission rate in some countries is exceptionally high compared with others, and the number of infected patients is increasing gradually [36]. Chronologically, the first virus was obtained from a patient on 7 January 2020. In response, the sequence of the viral genome was published by Chinese scientists on 10 January 2020, on Global Initiative on Sharing All Influenza Data (GISAID) [37]. In China, five patients were admitted to the hospital between 18 and 29 December 2019, out of which one patient expired [38].

Additionally, 41 more patients were admitted to the hospitals by 2 January 2020. The diagnosis of these patients confirmed COVID-19. Thailand was the first country that was infected after China, and the first case was reported in Thailand on 13 January 2020, in a patient who traveled to Thailand. Five hundred and seventy-one new cases of COVID-19 were reported by 22 January 2020, in 25 provinces of China [39]. National Health Commission of China (NHC) confirmed the first 17 deaths due to COVID-19 by 22 January 2020. On 25 January 2020, there were a total of 1975 infected patients in China, out of which 56 expired [40]. According to another report from 30 January 2020, a total of 7734 cases were reported in China, and 90 cases were confirmed from other countries including Vietnam, Nepal, Sri Lanka, Japan, Thailand, Republic of Korea, United States, United Arab Emirates, Malaysia, The Philippines, India, Australia, Canada, Cambodia, Finland, Singapore, France, Germany, and Taiwan [41].

The Chinese Center for Disease Control and Prevention reported 44,672 confirmed cases by 11 February 2020, in which 81% of deaths were seen in patients age over 60 years. Mortality rates in patients aged 70–79 and over 80 years were 8.0% and 14.8%, respectively [42,43]. On 28 February 2020, WHO reported 82,000 confirmed cases worldwide, and the outbreak reached 45 countries other than China. Following the rapid transmission course, 90,000 infected cases and 60 countries were dealing with the COVID-19 outbreak by 2 March 2020. Previously, it was thought that children were less susceptible to infection owing to a lack of evidence, but on 5 March 2020, Chinese studies concluded that children are as susceptible as adults. By 13 March 2020, Europe was declared as the epicenter of the outbreak by WHO. There were a greater number of reported cases in Europe as compared with the rest of the world. In Europe, Italy was the largest outbreak territory. The United States President declared the national emergency in the United States on 13 March 2020. By 27 March 2020, the total number of infected patients reached half a million, and pandemic spanned about 175 countries. The one million mark globally was reached on 2 April 2020. The next million cases were reported in less than two weeks, and there were 2 million cases worldwide on 15 April 2020, with the United States being on the top of the list, followed by Italy and Spain [44]. Currently, there are almost 21 million cases with 752,225 deaths around the globe, accessed on August 13, 2020 (https://coronavirus.jhu.edu/map.html).

Rapid transmission of COVID-19 reflects the high transmissibility and reproductive number (R_o_). Chinese reports show a R_o_ value of 2.2–2.7, meaning the infected cases double every 6–7 days [45]. The rate of transmission was inconceivably high, so several medical facilities were established, and guidelines were reported by the CDC and the WHO to manage the escalating disease [46].

Another parameter for understanding the severity and prognosis of infection is the case fatality ratio (CFR), which is calculated by dividing the number of expired patients by the total number of diagnosed patients, multiplied by 100. According to the WHO situation report-185 of COVID-19, the overall CFR was 5.12 as of 23 July 2020. A remarkable difference in CFR was noted in some countries presented in Table 1. The exact cause for this variability is still a topic of investigation in the scientific community. CFR is useful in estimating the risk of death within a population owing to a specific disease. Data suggest increased mortality with advanced age has a well-known impact on the prognosis of the disease. The median age of any population reflects the variation in fatality rates [47]. One of the Chinese reports revealed that the mortality rate could be 3% high in geriatrics, especially over 80 [48].

## 5. Pathogenesis

SARS-CoV-2 targets the lower respiratory tract and causes mild to severe symptoms. COVID-19 patients show significant clinical symptoms, including non-productive cough, myalgia, reduced leukocyte, dyspnea, fever, fatigue, and pneumonia. These symptoms are also shown by the patients infected by SARS-CoV and MERS-CoV [49]. Although the pathogenesis of novel coronavirus disease is not fully understood, the similarity of novel coronavirus with SARS-CoV can provide significant information about the pathogenesis of COVID-19 infection. The body shows aggressive inflammatory responses after viral entry, and it may cause severe damage to airways. The pathogenicity is increased by a large number of viral copies replicated in the cell. The life cycle of SARS-CoV-2 is divided into four stages: (i) entry of the virus, (ii) protein expression, (iii) transcription, and (iv) release of the virus from the cell [50].

Coronavirus enters the cell by its sole determinant viral S-protein and undergoes several steps to accomplish the replication. While S-protein is divided into two domains, S1 and S2 domain, the S1 domain is responsible for binding with the receptor, and the S2 domain accounts for the fusion of the viral membrane with the cell membrane [51]. The viral spike glycoproteins attach themselves to the ACE2 receptor of the cell, for both viruses SARS-CoV [52] and SARS-CoV-2 [53]. Initially, the virus binds to the cell receptor and enters the cell through the process of membrane fusion of the virus and plasma membrane [54]. Belouzard et al. [55] showed that the proteolytic processes occur in the S2 domain, which accounts for fusion and infection. After the viral entry, the RNA genome of the virus is released in the cytoplasm and then undergoes translation, followed by transcription, through which the virus continues to replicate [56]. After the formation of viral proteins by translation, these new proteins are inserted into the endoplasmic reticulum or Golgi apparatus. When viral RNA is combined with the proteins, the nucleocapsid is formed. Finally, the newly formed viruses enclosed in the vesicles are released by exocytosis [56]. The viral release marks the time when infected patients start displaying significant symptoms and laboratory values. Higher leukocyte count, respiratory problems, and exaggerated pro-inflammatory cytokines are exhibited by COVID-19 patients. In a case report, a patient presented with fever for the past two days displayed abnormal breathing sounds and a temperature of 39.0 °C. The real time-polymerase chain reaction (RT-PCR) conducted on the sputum culture showed a positive result for COVID-19 and confirmed the disease [57].

**Figure 2:**
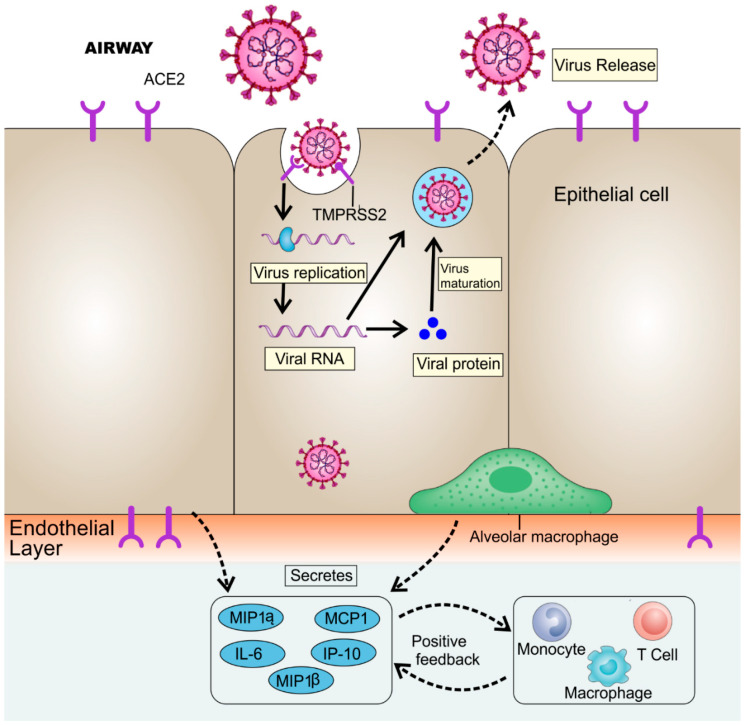
Inflammatory responses during severe acute respiratory syndrome coronavirus (SARS-CoV-2) infection. SARS-CoV-2 attaches to the ACE2 receptor, and the spike protein is cleaved by the type II transmembrane serine protease (TMPRSS2), resulting in viral replication. Mature viruses are released from the cell by exocytosis. In the dysfunctional immune response, cells may undergo pyroptosis and release adenosine triphosphate (ATP), nucleic acids, and ASC oligomer. These impaired-associated molecular forms can be detected by the neighboring epithelial cells and other alveolar macrophages. In response, the pro-inflammatory cytokines are released along with chemokines, including IL-6, IP-10, macrophage inflammatory protein 1α (MIP1β), MCP1, and MIP1α. These released proteins draw the monocytes, T cells, and lymphocytes to the inflammatory site, causing exaggerated inflammation at the site of action. This exaggerated response damages the lung’s infrastructure. This cytokine storm can move to other organs and may damages the other organs as well.

The laboratory analysis showed a leukocyte count of 2.91 × 10^9^ cells/L, of which neutrophils were 70%. Further, the C-reactive protein was 16.16 mg/L (normal range = 0–10 mg/L). The erythrocyte sedimentation rate (ESR) was also high with D-dimer. The primary pathogenesis of the nCoV is associated with respiratory infections, pneumonia, ground-glass opacities (GGOs), acute cardiac injury, ARDS, and RNAaemia [38]. Additionally, high blood values of chemokines and cytokines were also observed in patients, including IL1-β, IL7, IL8, IL9, IL10, IL1RA basic FGF2, GMCSF, IFNγ, IP10, GCSF MCP1, MIP1α, MIP1β, PDGFB, and TNFα, as shown in Figure 2. Critical patients showed a high value of pro-inflammatory cytokines as a cytokine storm, including IL2, IL7, IL10, GCSF, IP10, MCP1, MIP1α, GCSF, and TNFα, which exaggerated the severity of the infection [58]. This exaggerated immune response destroys the lung’s infrastructure [59].

Current models give information about the three stages of the immune response. The first stage involves the early activation and persuasive interferon response for clearing the virus. The second stage exhibits the delayed response of interferon that may lead to tissue damage. The third stage leads to hyper-inflammation, followed by exaggerated macrophage activation, resulting in fibrosis dysregulation of tissue repair processes [59]. For each step, potential therapeutic options can be developed. Some are under clinical trials to evaluate their efficacy along with safety. Initially, the virus attaches to the ACE2 receptor. TMPRSS2 is responsible for the viral protein cleavage. Protease inhibitors can be designed and developed to prevent spike protein cleavage. Blocking the viral fusion through either TMPRSS2 or receptor ACE2 can prevent the infection. For removing pro-inflammatory cytokines, several novel models have been proposed in which the blood of a COVID-19 patient is passed through customized columns that recognize and trap the pro-inflammatory cytokines, and then the purified blood is administered back to the patient [60]. ARDS seen in several COVID-19 patients may lead to secondary infections, and respiratory failure leads to death in 79% of critical cases. Furthermore, the cytokine storm may lead to sepsis and cause death in 28% of critical cases [61]. Scientists are trying to design therapeutics to prevent this exaggerated immune response responsible for the enhanced severity of the infection [60,62].

As discussed earlier, ACE2 is the receptor for SARS-CoV-2. It has been reported that the involvement of ACE2 receptors is also related to the renin–angiotensin system (RAS) in 2019-nCoV pathogenesis [63]. Very recently, a meta-analysis was conducted to get insight into the relationship between the prevalence of COVID-19 disease and the genetic differences in the genes involved in the RAS system [63]. The results of the study suggest that the increase of the I/D allele frequency ratio significantly increases the recovery rate (point estimate: 0.48, confidence interval (CI) 95%: 0.05–0.91, *p* = 0.027), but the I/D allele frequency ratio has no significant difference in the case of death rate (point estimate: 1.74, CI 95%: 4.5–1.04, *p* = 0.22). The I/D allele ratio of ACE receptors varies significantly across world regions, accounting for the different recovery rates across regions, but it also raises concerns that ethnic and genetic differences can impact the effectiveness of currently investigated RAS-associated medications in different regions [63].

## 6. Clinical Features

SARS-CoV-2 represents an incubation period of 6.4 days, fluctuating between 2.1 and 11.1 days with possible asymptomatic transmission [64]. Infection is manifested after the incubation period. The period from the commencement of disease leading to death fluctuates between 6 and 41 days with an average of 14 days. The age of patient and condition of the immune system are the sole determinants of this period. Persons with a weaker immune system are more susceptible to infection. This period is shorter in geriatrics >60 years, as older patients have a weaker immune system [65]. The most common symptoms of COVID-19 are dry cough, myalgia, diarrhea, headache, and high-grade fever [66]; fever is reported in more than 90% of cases, cough in 75%, and dyspnea in 50% of the patients [8]. Fewer patients may also develop kidney damage, ARDS, septic shock, and acute cardiac injury requiring hospitalization [67]. The clinical symptoms are presented in Table 2.

**Figure 3:**
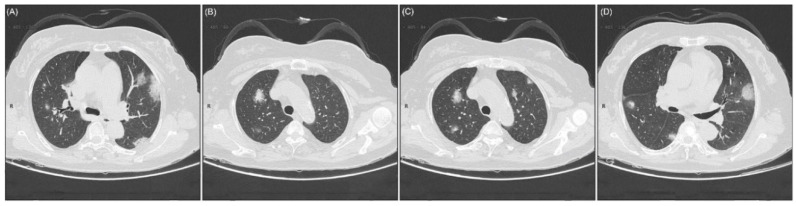
Atypical CT imaging of a 56-year-old female presented with a history of 3 days of persistent fever. Laboratory findings: reduced total protein level (54.0 g/L), reduced albumin value (35.5 g/L), low value of globulin (18.5 g/L), white blood cells (WBCs) (4.87 × 10^9^/L), lymphocytes (0.49 × 10^9^/ L), and decreased level of eosinophil (0 × 10^9^/L). Imaging inspection: (**A**) A lateral section of the center of right lung lobe covered GGO. (**B**) Patchy GGO in the upper part of the right lung with sporadic consolidated lesions. (**C**) GGO in both lungs with sporadic consolidation lesions. (**D**) Uneven GGO in the middle and dorsal section of the right lung. Adopted from Kui et al. through open-access license [69].

Mutual laboratory findings include lymphopenia and leucopenia. These findings were common and observed in a large number of cases [68]. Chest computed tomography (CT) scan showed clinical features including pneumonia and other abnormal features, for instance, RNAaemia, acute cardiac injury, and occurrence of GGO, that set the path towards death. In several cases, multiple marginal GGOs were observed in both lungs, which expectedly triggers the systemic and localized immune reaction, causing severe inflammation [65]. Atypical CT scan showed single, multiple solid, and consolidated nodules in the middle of the lobe of right or left or both lungs, fenced by GGOs in the number of COVID-19 patients (Figure 3) [69].

Although there are some similarities, the symptoms manifested by earlier coronaviruses are not precisely similar to those of COVID-19. For instance, the distinctive features of COVID-19 are fever, fatigue, and dry cough. In contrast, upper respiratory tract symptoms like rhinorrhea, sore throat, and sneezing are uncommon, which were commonly observed in previous coronavirus infections. Moreover, COVID-19 patients present with intestinal symptoms like diarrhea, which were less prevalent in MERS-CoV and SARS-CoV infections [70].

In addition, patients may also report anosmia and dysgeusia as early symptoms of COVID-19 [71]. Anosmia (loss of sense of smell) and dysgeusia (alteration of the sense of taste) are also associated with COVID-19 patients. The American Academy of Otolaryngology-Head and Neck Surgery developed the COVID-19 Anosmia Reporting Tool for clinicians to conduct a pilot study. This tool allows clinicians to submit the anosmia and dysgeusia associated COVID-19 patients. They analyzed the first 237 entries and revealed that anosmia was observed in 73% of patients prior to the diagnosis of COVID-19 and was the early symptom in 26.6% of COVID-19 patients. Conclusively, this study suggested that anosmia can be the representative symptom of COVID-19 [72,73].

## 7. Diagnosis

The diagnosis and treatment program for COVID-19 was formulated by NHC of China [69,74] and was previously suggested by WHO for SARS and MERS [75,76,77,78]. The specific diagnostic measures have been implemented for suspected as well as confirmed cases: a patient with a minimum of two clinical symptoms and at least one exposure history is accounted as a suspected case. The suspected case should show at least three clinical symptoms if there is not any clear exposure history.

The serology testing for COVID-19 is helpful and provides the basis for diagnosis. In the case of the unavailability of molecular testing, the serological test provides a means to triage the suspected cases of COVID-19. A fast-serological test with specific performance features is highly important to avoid missing factual cases of COVID-19. A positive test for IgM or IgG antibody strongly indicates SARS-CoV-2 infection. This approach is extremely effective in individuals 5–10 days after the onset of symptoms [79]. IgG or IgM positive patients are quarantined, and those who need critical care are referred to the hospital. Those with a negative serological antibody test have a swab collected for molecular testing. The experience in China has shown that the use of a serological antibody test can improve the sensitivity of COVID-19 case detection [80]. This approach allowed to test a huge number of symptomatic individuals rapidly in the community, minimizing the waiting time for molecular testing and preventing the health-management system from being overwhelmed [79].

Subsequently, when nucleic acids of SARS-CoV-2 by RT-PCR results come back positive, it is accounted for as a confirmed case [76]. Meanwhile, a CT scan of the chest provides confirmed diagnostic evidence of COVID-19 disease as it has greater sensitivity and high specificity. Thus, the chest CT has been recognized as a significant indicator for COVID-19 diagnosis in different epidemic areas [15,31,81]. It can be said that the chest CT is an essential procedure for the timely detection and management of this infection. However, it should be noted that a patient with an RT-PCR may have a negative chest CT at the time of admission [82]. In this context, a study compared three RT-PCR analyses, targeting the RNA-dependent RNA polymerase RdRp/Helicase, spike, and nucleocapsid genes of the virus with testified RdRp-P2 assay, which is extensively used in more than 30 European countries. According to this study, RdRp/Hel is more specific and gives accurate results for SARS-CoV-2. Moreover, the RdRp/helicase assay of COVID-19 does not cross-react with other types of coronaviruses and respiratory microorganisms in the cellular medium. In contrast, the RdRp-P2 assay, reported for previous coronaviruses, reacts with the pathogens of the respiratory tract in the cell culture. Thus, the COVID-19-RdRp/Hel can be considered sensitive and specific, and this information helps fortify the diagnosis of COVID-19 [83].

Laboratory findings can be helpful in predicting COVID-19 cases with positive RT-PCR. ALT, C-reactive protein, neutrophils, AST, and lymphocytes counts have very good accuracy in predicting cases with positive RT-PCR for COVID-19 patients [84]. A study suggested that the number and percentage of white blood cells (WBCs), lymphocytes, and neutrophils were significantly different between positive and negative RT-PCR cases for COVID-19 patients [85]. Another cohort study suggested that lymphopenia (low levels of lymphocytes) occurred in 80% of COVID-19 patients [86]. An increased level of AST and ALT was also noted in severely affected patients [87]. High levels of LDH, C-reactive protein, neutrophils, and erythrocyte sedimentation rate (ESR) were also notable in COVID-19 patients [88]. Thus, these laboratory findings have great importance in terms of predicting COVID-19 cases with positive RT-PCR [84].

## 8. Treatment

Currently, no specific and sensitive therapeutic regimen is available for the treatment of COVID-19. However, the primary step is isolation to prevent any type of contact that later may cause transmission of the disease. If the patient experiences any mild symptoms, it should be managed at their residence with proper counseling to patients about the critical signs and symptoms. The primary management protocols include the management of cough and fever [89]. First-line therapy to manage the fever is acetaminophen, while expectorants such as guaifenesin are used for non-productive cough [90]. Patients with SARS, hypoxemia, respiratory distress or shock should be treated by the administration of instant oxygen therapy. The frequency of oxygen therapy should be 5 L/min to obtain SpO_2_ of ≥92–95% in pregnant women, and ≥90% in adults and children [91]. Antiviral therapy is also being tried to manage the infection [92].

Critical patients show an unhealthy immune response that eventually causes hyper-inflammation. This exaggerated immune response should be controlled and checked by different approaches and procedures. As discussed earlier, inflammatory cytokines are released at the site of infection and cause infiltration of lungs. Clinical trials are underway to identify the benefits of blocking cytokine storm targeting IL-1β and IL-6, as shown in Table 3.

Interfering cytokine storm signaling can prevent hyper-inflammation, thereby reducing infection. Current evidence shows that the overproduction of macrophages linked to monocytes accumulates in tissue that can cause lung infiltration. For instance, it is found that CCR2 activation contributes to the accumulation of monocytes in the tissue, causing severe inflammation. So monocytes’ accumulation can be reduced by blocking the CCR2, resulting in minimizing hyper-inflammation [96]. Different clinical trials are underway to reduce the hyper-inflammation by JAK inhibitors [97]. Other relevant clinical trials are ongoing to evaluate the blocking of myeloid-derived cytokines, for example, TNF [98] and GM–CSF. By targeting this cytokine signaling, the hyper-inflammation can be reduced [99].

Remdesivir is a broad-spectrum antiviral prodrug, which is a nucleotide analog and is metabolized in the cell to adenosine triphosphate analog, which inhibits the viral RNA polymerases. Remdesivir is the drug of choice against several viral families, including Ebola, SARS-CoV, and MERS-CoV. Remdesivir is being used for prophylactic and therapeutic purposes and has shown remarkable efficacy in the non-clinical model of coronaviruses [100,101]. It has also shown promising activity against SARS-CoV-2 [102,103]. A large clinical trial of remdesivir is currently underway for patients with mild to moderate symptoms [104]. The critically ill patients treated with amicable use of remdesivir showed better efficacy, and the clinical condition of the patients was improved [105]. On 1 May 2020, the Food and Drug Administration (FDA) of the USA authorized the emergency use of remdesivir for the treatment of COVID-19 [106]. The recent version of treatment guidelines by NHC recommends the use of lopinavir/ritonavir and IFN-α. The first version of treatment guidelines was issued on 15 January 2020, later revised five times, and the 6th edition is the most recent one that was issued on 18 February 2020. Lopinavir/ritonavir, ribavirin, and IFN-α were added in the 5th edition of treatment guidelines for combating COVID-19. Later, arbidol and chloroquine phosphate have also been added in the 6th edition, as these drugs show better clinical efficacy. Potential drugs included in the 6th edition of treatment guidelines by NHC of China, as shown in Table 4, presented promising therapeutic effects [107]. Although the recent study was an open-label, individually randomized, controlled trial representing patients admitted in hospitals, treated with lopinavir/ritonavir (400 mg/100 mg), no clinical improvement was observed by this treatment beyond standard care [108]. On the basis of the guidelines, ritonavir is used as a booster to enhance the efficacy of lopinavir. These medications are prescribed to be taken for a short period of time (<2 weeks), suggesting their acute response.

Hydroxychloroquine, lopinavir/ritonavir, chloroquine, and darunavir/cobicistat are being used for patients with severe conditions. Interferon-beta B1 is extensively used for critical patients [110]. Another drug, oseltamivir, is being used in China by the medical personnel for suspected patients, but there is a lack of evidence on the specific activity of oseltamivir against COVID-19. A 54-year-old female patient from Wuhan city presented with fever for the past two days. Two tests were conducted, the first test was not positive, but the second test confirmed the disease. Meanwhile, this woman was treated with oseltamivir for three days, and her chest CT indicated reminiscent improvement in the density of GGO [90].

Patients with vigorous immune reactions can be treated with glucocorticoids such as methylprednisolone, which is used at 1–2 mg/kg/day dose in children and 25–150 mg/day in adults. It is believed to reduce the prognosis of the disease in patients with impaired oxygen index, that is, less than 300 mm Hg, limited to 5 days [69]. For patients with premature disease stage, without hypoxia, use of corticosteroids should be avoided. However, in critical situations where mechanical ventilation is mandatory, anti-inflammatory therapy is possibly useful and can be employed [59]. A glucocorticoids-based drug dexamethasone may be employed against COVID-19. Dexamethasone is a synthetic corticosteroid approved by the FDA in 1958 as a broad-spectrum immunosuppressor, which is about 30 times as active as cortisone and possesses a longer duration of action (2–3 days). Dexamethasone may not only limit the production and damaging effect of the cytokines, but may also inhibit the protective function of T cells and reduce the ability of B cells to synthesize antibodies [111]. Dexamethasone may be a useful agent for short-term therapy in severe intubated COVID-19 patients [112]. A recent controlled open-label, randomized trial of dexamethasone in hospitalized patients, concluded that the use of dexamethasone with the aid of mechanical ventilation or oxygen alone resulted in lower mortality at the dose of 6 mg once daily for 10 days. However, dexamethasone was not effective in those patients who were not receiving respiratory support [113]. Dexamethasone is on an essential medicines list recommended by the World Health Organization and is readily accessible worldwide at a low cost. Guidelines issued by the U.K.’s chief medical officers and by the National Institutes of Health in the United States have already been updated to recommend the use of glucocorticoids such as dexamethasone in hospitalized patients with COVID-19 [114,115].

As discussed earlier, a variety of drugs are recommended and tested in non-clinical models; among them, penciclovir, ribavirin, and favipiravir require high doses to decrease the infection and are supposed to be less effective [90]. Chloroquine and its derivative hydroxychloroquine are considered to be effective against COVID-19 and are currently used in Chinese patients to reduce viral infection. Potential drug therapy for COVID-19 is listed in Table 4 to gain a better understanding of treatment [116]. An open-label, non-randomized clinical trial showed that hydroxychloroquine (600 mg/day) treatment is magnificently related to a decreased rate of viral replication in patients, and its effectiveness is reinforced by azithromycin (500 mg/day) [116]. Hydroxychloroquine showed superior sensitivity over chloroquine against COVID-19 [117]. Contrary to the previous results, at the time of publication, an open-label, randomized controlled clinical trial showed that the use of hydroxychloroquine alone or with azithromycin did not improve the clinical status of hospitalized patients with mild to moderate COVID-19 as compared with standard care [118].

According to many reports, the COVID-19 patients are at high risk for venous thromboembolism, a term that associates deep vein thrombosis (DVT) [119]. In this context, patients are treated prophylactically by giving low molecular weight heparin (LMWH), when multiple risk factors exist. Hence, hospitalized COVID-19 patients are generally treated with higher doses of LMWH than recommended for thromboprophylaxis [120]. A recent document by the Italian Drug Agency (AIFA) suggested the use of 80 to 100 mg enoxaparin daily, instead of the usual 40 mg, while in some hospitals, even higher, up to full anticoagulant doses of LMWH or unfractionated heparin are used [121].

SARS-CoV-2 targets the ACE2 receptor via spike-like protein, so it is an efficient target for the drugs, including chloroquine, promazine, and emodine, to prevent the viral attachment with the surface of the host cell [122]. S protein priming is the next precarious phase of viral entry into the host cell, where it is required to be cleaved by intracellular proteases including TMPRSS2, cathepsins, and furin for the fusion of viral membrane with the cell membrane of the host. S-protein priming by TMPRSS2 is the most crucial phase for the prognosis of infection into the host, so the viral infection can be controlled with the use of protease inhibitors, including camostat and nafamostat, which target TMPRSS2, and thereby inhibit the viral fusion [123]. The immune system represents the defensive mechanism in attacking the virus; major immune cells include T-lymphocytes and natural killer cells [122]. Apart from antiviral therapy, the use of antibiotics can be used for the associated co-infections, community-acquired, or hospital-acquired pneumonia, and it depends on the clinical results of the patients. These broad-spectrum antibiotics, including azithromycin and fluoroquinolones, can be given as empirical therapy to cover all attainable pathogens, continuing the therapy until all pathogenic bacteria are cleared from the body. The combination therapy of azithromycin with hydroxychloroquine showed significant activity against COVID-19 infection [124]. Table 5 delineates the potential therapeutics against COVID-19.

Some researchers recommend the use of vitamin C supplements that may help to treat the clinical symptoms of the patient by potentially activating the immune system. These supplements help to reduce the severity of pneumonia; however, this requires more clinical evaluation [131]. Supplements of vitamin D_3_ can also be useful for combating the infection. While vitamin D can activate the innate immune system, its decreased value indicates enhanced autoimmunity and susceptibility towards infection. Grant et al. indicated the use of vitamin D in decreasing the probability of respiratory infections caused by COVID-19. As vitamin D induces the antimicrobial peptides such as defensins and cathelicidins that eventually interfere with the viral growth and pro-inflammatory cytokines, more evaluation is needed by randomized controlled trials on a large number of individuals to reach to a conclusion [132].

Presently, no vaccine exists against COVID-19; however, several clinical trials are underway, as discussed in Table 6. Scientific research is ongoing by various medical institutes and companies to produce effective vaccines all over the world that can combat COVID-19.

A most recent study published in Lancet claims the production of a vaccine named PittCoVacc developed by the University of Pittsburgh School of Medicine. PittCoVacc uses S-protein, which provides protection by the induction of specific antibodies in the body. Researchers are conducting different analyses on the vaccine, and they are planning a series of tests in humans in the coming months [129]. Another vaccine named mRNA-1273 is being developed by the National Institute of Allergy and Infectious Disease researchers combined with biotechnology institution Moderna. In this case, mRNA is enclosed in lipid nanoparticles that encode the S-protein of the virus, and then it takes the antigen into the body. In response to antigen, specific antibodies are produced that provide protection against SARS-CoV-2. The clinical trial of the mRNA vaccine is underway, while scientists are working together to evaluate the pathogenesis and clinical features for combating the disease by approaching a series of therapeutic options and development of other vaccines [133].

University of Oxford, United Kingdom, conducted a randomized clinical trial of the COVID-19 vaccine named ChAdOx1 nCoV-19 vaccine. The estimated participants in this study were about 10,260, and this vaccine was administered intramuscularly. Fortunately, this vaccine appeared to be most effective in participants showing an effective response and has already entered into second and third clinical trials (clinicaltrials.gov).

Finally, there is an emerging concern that patients recovering from COVID-19 may be at risk of reinfection. To address this concern, Bao et al. investigated acquired immunity to SARS-CoV-2 in rhesus macaques. This study published recently suggests that immunity acquired following primary infection with SARS-CoV-2 may protect upon subsequent exposure to the virus [134]. However, more studies are needed to reach a definitive conclusion.

## 9. Prevention and Control

Several methods have been implemented based on actual infection control and segregation to control the transmission of COVID-19. WHO and CDC published guidelines for the prevention and control of the infection. For instance, individuals should wash their hands with alcohol-based sanitizers on a routine basis. Touching of nose, mouth, and eyes should be avoided as they are the critical part for holding the infection. Moreover, a distance of approximately of 1 m from those who are sneezing and coughing should be maintained. People should uphold respiratory hygiene, such as covering nose and mouth during coughing. Individuals should wear a mask while meeting with other persons or going outside. Individuals are supposed to sanitize their workplaces [135]. Medical teams are supposed to cover their nose and mouth by licensed N95 masks. In some areas, the availability of the N95 mask is limited; in this case, the use of surgical masks while treating suspected and confirmed patients should be ensured. Proper use and disposal of the surgical mask is necessary to minimize the risk of transmission [136,137]. As there are clinical trials of vaccines underway, for now, the most effective prevention for the entire population is to minimize the exposure to the virus. Center for Disease Control, USA, WHO, and the Chinese CDC have provided proper guidelines to enhance awareness among the population about the prevention of COVID-19. The main emphasis of the guidelines includes awareness on how to wear a face mask, appropriate hand washing techniques, preventive measures at home and workplaces, and disinfection procedures and protocols. Furthermore, the guidelines are available on the treatment, prevention, and control of COVID-19. The guidelines also endorse ways of minimizing the fear of COVID-19 among the overall population [138]. Considering all the nonpharmaceutical measures assist the maintenance of the public outcomes of the pandemic to be in control, because of several financial reasons, the numbers are again elevating. There are multiple steps involved in preparation for personal protection equipment (PPE), including identifying PPE and to ensure a proper gown size, performing hand hygiene using hand sanitizer, wearing proper masks, put on face shield or goggles, and gloves in areas that could be necessary. The guidelines could be different based on the profession, location, and severity of the disease.

## 10. Conclusions

This review provides insight into various aspects of COVID-19, including disease pathophysiology, its clinical representation, mechanism of drugs actions, and impacted population. However, the major concentration of this report was to emphasize potential therapeutics and provide all essential information related to it in one document. On the basis of the class of drugs, multiple classes of drugs have been shown to work primarily to stabilize the patient and, secondly, target the virus for treatment.

The first class of drugs is anti-inflammatory ones that deal with cytokines signaling through targeting various receptors such as IL-6. The major outcome of the treatment is to reduce the inflammation during the therapy. The second group of medications is classic orally administered drugs that have been used for antiviral or anti-parasite ones such as chloroquine phosphate and lopinavir/ritonavir. Among them, remdesivir has been approved by FDA for the emergency room treatment of COVID-19, suggesting its promising potency. In addition to all the mentioned medications, corticosteroids are used to assist with reducing the pro-inflammatory effect. However, owing to their unfavorable side effect profile, their use should be limited to a short period of time in critical patients.

It needs to be emphasized that, among all tested drugs, antiviral compounds such as remdesivir inhibit RNA transcription, leading to reduced viral load. This class of drugs is potentially the most promising tool with which to treat COVID-19 patients. Thus, although there is no solid evidence available to prove their long-term clinical outcomes as of this day, drugs targeting viral RNA are the most promising tools in this battle. Further investigations are required to test a library of synthesized antiviral agents to obtain the most potent candidate to be used in clinics. The ongoing trials of the vaccine will be a hope to provide a vaccine displaying efficacy without side effects to overcome the COVID-19 pandemic to humankind.

## Figures and Tables

**Table 1:** Case fatality ratio of Coronavirus Disease 2019 (COVID-19) in major countries of the world *. CFR, case fatality ratio.

Territory	Total Confirmed Cases	Total Deaths	CFR
France	167,456	30,060	17.95
Belgium	296,381	45,501	15.35
Italy	64,534	9808	15.19
United Kingdom	245,032	35,082	14.32
Netherlands	52,241	6139	11.75
Spain	267,551	28,426	10.62
Sweden	111,697	8862	7.93
Canada	78,504	5667	7.21
Ireland	25,819	1754	6.79
Brazil	86,361	4655	5.39
United States of America	281,413	14,853	5.28
China	33,796	1692	5.01
Switzerland	203,368	9101	4.47
Japan	2,159,654	81,487	3.77
Iran	27,029	990	3.66
Germany	3,868,453	141,479	3.65
Austria	20,038	711	3.55
Turkey	222,402	5545	2.49
Australia	12,896	128	0.99
Saudi Arabia	258,156	2601	1.00
Grand total	8,482,781	434,541	5.12

* World Health Organization (2020). COVID-19 Situation Report-185 (Accessed on 23 July 2020).

**Table 2:** Clinical symptoms integrated with the severity of COVID-19 [48,66].

Clinical Type	Symptoms
Mild disease	No signs of pneumonia; it happened in 81% of patients.
Severe disease	Oxygen saturation of blood ≤93%, infiltration of lungs ≥50% within one or two days. The ratio of PaO2/FiO2 (oxygen partial pressure/fraction of inspired oxygen) <300. GGO (ground glass opacities): 14% of cases acquired severe symptoms.
Critical disease	Failure of the respiratory system, septic shock, concerted organ dysfunction, or multiple organ failure (MOF); it happened in 5% of patients.

**Table 3:** Potential therapeutic targets associated with macrophage activation under clinical trials [93,94,95].

Drug Name	Drug Type	Target	Role of Target	Clinical Trial Number
Tocilizumab	Anti-IL-6 receptor	IL-6 signaling process	Pro-inflammatory	NCT04306705; NCT04346355; NCT04320615; NCT04317092; NCT04335071; ^a^ NCT04331808
No drug approved	Various	TLR4–TRIF signaling	Pro-inflammatory	NA
Emapalumab	Anti-IFNγ	IFNγ	Pro-inflammatory	NCT04324021
Tofacitinib	JAK1/JAK3 inhibitor	JAK-STAT signaling	Pathway mediates cytokine signaling	^a^ NCT04340232; ^a^ NCT04320277
Cenicriviroc (not approved)	CCR2 and CCR5 antagonist	CCR2	Promotes monocyte recruitment in tissues	Clinical trial in progress
Sargramostim	GM-CSF	GM-CSF signaling	Pro- inflammatory; repairing lung tissues	NCT04326920
Fliximab; adalimumab; golimumab	Anti-TNF	TNF signaling	Pro- inflammatory	NA
Anakinra	IL-1 receptor antagonist	IL-1β signaling	Pro-inflammatory	NCT04339712; NCT04324021; NCT04330638; ^a^ NCT04341584

Data from ClinicalTrials.gov. IFNγ, interferon-γ; JAK, Janus kinase; CCR2, CC chemokine receptor 2; CCR5, CC chemokine receptor 5; STAT, signal transducer and activator of transcription; GM-CSF, granulocyte-macrophage colony-stimulating factor; NA, not applicable; TLR, Toll-like receptor; ^a^ clinical trials not yet recruiting at time of publication.

**Table 4:** Antivirals included in the treatment guidelines issued by NHC of China for COVID-19, version 6 [107,109]. NHC, National Health Commission; t.i.d, three times a day; b.i.d, two times a day.

Drug Regimen	Dosage	Route of Administration	Length of Therapy
Ribavirin	500 mg, t.i.d, combination therapy with lopinavir/ritonavir	IV infusion	≤10 days
Chloroquine phosphate	500 mg one time, b.i.d	Orally	≤10 days
Arbidol	200 mg, one time, t.i.d	Orally	≤10 days
Lopinavir/ritonavir	200 mg/50 mg, dosage form: capsule, two capsules one time, b.i.d	Orally	≤10 days
IFN-α	5 million U dose one time, b.i.d	Vapor inhalation	≤10 days

**Table 5:** Potential therapeutics against SARS-CoV-2 (COVID-19) [68,105,112,116,125,126,127,128,129,130]. IFNs, interferons.

Drug	Type	Mechanism	Recommendation
Remdesivir	Antiviral	Remdesivir targets the viral RNA polymerases to inhibit the replication of the virus, previously used for Ebola	Clinical trials have been conducted
Favipiravir	Antiviral	Favipiravir interacts with viral RNA polymerase, thus inhibiting viral replication	Efficacy has been proven
Convalescent plasma	Antiviral	Convalescent plasma obtained from the cured ones provides protective antibodies against SARS-CoV-2	Clinical trials have been conducted
Chloroquine Hydroxychloroquine	Anti-malaria	Inhibition of endosomal acidified fusion and also have an anti-inflammatory effect	Efficacy has been proven
IFNs	Immuno-enhancer	IFNs interfere with the viral RNA transcription and translation of protein, and thus inhibit viral replication	Clinical trials have been conducted, and efficacy has been proven
Lopinavir/ritonavir	Antiviral	It inhibits the protease enzyme, thereby preventing the cleavage of polyproteins, and it produces immature particles.	Clinical trials underway
Corticosteroids	Corticosteroids	Corticosteroids diminish pro-inflammatory cytokines and show an anti-fibrotic property	Controversial state
Dexamethasone	Glucocorticoids	Dexamethasone inhibits the protective function of T cells and reduces the ability of B cells to synthesize antibodies, as well as limits the damaging effects of cytokines	Clinical trials underway
Vitamin C	Nutritional supportive management	Vitamin C strengthens the immunity response by increasing IFN production and enhancing the capability of phagocytosis by neutrophils	Clinical trials have been conducted
Zinc	Nutritional supportive management	Zinc is mandatory for the efficient activity of the immune system and also has antiviral property, as it also enhances the therapeutic activity of hydroxychloroquine	Clinical trials have been conducted
Enoxaparin	Low molecular weight heparin (LMWH), anticoagulant	Enoxaparin showed its anticoagulant activity and is used in thromboprophylaxis in COVID-19 patients	Clinical trials underway

**Table 6:** Development of vaccines for COVID-19 that are under clinical trials.

Vaccine	Allocation	Title of Study	Location	Clinical Trial Number
BCG vaccine	Randomized	Reducing Health Care Workers Absenteeism in Covid-19 Pandemic Through BCG Vaccine (BCG-CORONA)	Multiple sites in the Netherlands	NCT04328441
SARS-CoV-2 inactivated vaccine	Randomized	A Randomized, Double-Blinded, Placebo-Controlled, Phase Ⅰ/Ⅱ Clinical Trial, to Evaluate the Safety and Immunogenicity of the SARS-CoV-2 Inactivated Vaccine in Healthy Adults	Suining County Center for Disease Control and Prevention in China	NCT04352608
mRNA-1273	Non-Randomized	Safety and Immunogenicity Study of 2019-nCoV Vaccine (mRNA-1273) for Prophylaxis of SARS-CoV-2 Infection (COVID-19)	Multiple sites in America	NCT04283461
LV-SMENP-DC vaccine and antigen-specific CTLs	NA	Phase I/II Multicenter Trial of Lentiviral Minigene Vaccine (LV-SMENP) of Covid-19 Coronavirus	Shenzhen Geno-immune Medical Institute Shenzhen, Guangdong, China	NCT04276896
bacTRL-Spike vaccine	Randomized	A Phase 1, Randomized, Observer-Blind, Placebo-Controlled Trial to Evaluate the Safety, Tolerability, and Immunogenicity of the bacTRL-Spike Oral Candidate Vaccine for the Prevention of COVID-19 in Healthy Adults	Canadian Center for Vaccinology Dalhousie University, IWK Health Centre	^a^ NCT04334980
ChAdOx1 nCoV-19 vaccine	Randomized	Phase 2/3 Study to Determine the Efficacy, Safety and Immunogenicity of the Candidate Coronavirus Disease (COVID-19) Vaccine ChAdOx1 nCoV-19 vaccine	Multiple sites in United Kingdom	NCT04400838
Inactivated SARS-CoV-2 Vaccine	Randomized	A Randomized, Double-blind, Placebo-controlled, Phase Ia/IIa Trial of an Inactivated SARS-CoV-2 vaccine in Healthy People Aged 18 to 59 Years	Multiple sites in China	NCT04412538

BCG, Bacillus Calmette–Guérin; ^a^ clinical trials not yet recruiting at the time of publication; data from clinical trials.gov.

## Abbreviations

SARS	severe acute respiratory syndrome
MERS	Middle Eastern respiratory syndrome
PHEIC	public health emergency of international concern
NHC	National Health Commission
CoV	coronavirus
ARDS	acute respiratory distress syndrome
ALI	acute lung injury
GISAID	global initiative on sharing all influenza data
GGO	ground-glass opacities
RT-PCR	real-time polymerase chain reaction
CCR2	CC chemokine receptor 2
CCL2	CC chemokine ligand 2
CCR5	CC chemokine receptor 5
TLC	thin-layer chromatography
JAK	Janus kinase
STAT	signal transducer and activator of transcription
GM-CSF	granulocyte-macrophage colony-stimulating factor
TLR	Toll-like receptor
BCG	Bacillus Calmette–Guérin

## References

[1] World Health Organization (2020). Coronavirus Disease (COVID-19) Situation Report 121, Data as Received by WHO from National Authorities by 10:00 CEST. https://www.who.int/docs/default-source/coronaviruse/situation-reports/20200520-covid-19-sitrep-121.pdf?sfvrsn=c4be2ec6_4.

[2] Liu Y., Gayle A.A., Wilder-Smith A., Rocklöv J. (2020). The reproductive number of COVID-19 is higher compared to SARS coronavirus. J. Travel Med..

[3] Gorbalenya A.E. (2020). Severe acute respiratory syndrome-related coronavirus—The species and its viruses, a statement of the Coronavirus Study Group. BioRxiv.

[4] Pillaiyar T., Meenakshisundaram S., Manickam M. (2020). Recent discovery and development of inhibitors targeting coronaviruses. Drug Discov. Today.

[5] Graham R.L., Donaldson E.F., Baric R.S. (2013). A decade after SARS: Strategies for controlling emerging coronaviruses. Nat. Rev. Genet..

[6] National Institute of Health COVID-19 Treatment Guidelines. https://covid19treatmentguidelines.nih.gov/introduction/.

[7] Di Gennaro F., Pizzol D., Marotta C., Antunes M., Racalbuto V., Veronese N., Smith L. (2020). Coronavirus diseases (COVID-19) current status and future perspectives: A narrative review. J. Environ. Res. Public Health.

[8] Ren L.-L., Wang Y.-M., Wu Z.-Q., Xiang Z.-C., Guo L., Xu T., Jiang Y.-Z., Xiong Y., Li Y.-J., Li H. (2020). Identification of a novel coronavirus causing severe pneumonia in human: A descriptive study. Chin. Med. J..

[9] World Health Organization (2020). Laboratory Testing for Coronavirus Disease 2019 (COVID-19) in Suspected Human Cases: Interim Guidance, 2 March 2020.

[10] Sohrabi C., Alsafi Z., O’Neill N., Khan M., Kerwan A., Al-Jabir A., Iosifidis C., Agha R. (2020). World Health Organization declares global emergency: A review of the 2019 novel coronavirus (COVID-19). Int. J. Surg..

[11] Zhou P., Yang X.-L., Wang X.-G., Hu B., Zhang L., Zhang W., Si H.-R., Zhu Y., Li B., Huang C.-L. (2020). A pneumonia outbreak associated with a new coronavirus of probable bat origin. Nature.

[12] Lee N., Hui D., Wu A., Chan P., Cameron P., Joynt G.M., Ahuja A., Yung M.Y., Leung C., To K. (2003). A major outbreak of severe acute respiratory syndrome in Hong Kong. N. Engl. J. Med..

[13] Zaki A.M., Van Boheemen S., Bestebroer T.M., Osterhaus A.D., Fouchier R.A. (2012). Isolation of a novel coronavirus from a man with pneumonia in Saudi Arabia. N. Engl. J. Med..

[14] Wang L.-F., Shi Z., Zhang S., Field H., Daszak P., Eaton B.T. (2006). Review of bats and SARS. Emerg. Infect. Dis..

[15] Bernheim A., Mei X., Huang M., Yang Y., Fayad Z.A., Zhang N., Diao K., Lin B., Zhu X., Li K. (2020). Chest CT findings in coronavirus disease-19 (COVID-19): Relationship to duration of infection. Radiology.

[16] Wang N., Shi X., Jiang L., Zhang S., Wang D., Tong P., Guo D., Fu L., Cui Y., Liu X. (2013). Structure of MERS-CoV spike receptor-binding domain complexed with human receptor DPP4. Cell Res..

[17] Zhong N., Zheng B., Li Y., Poon L., Xie Z., Chan K., Li P., Tan S., Chang Q., Xie J. (2003). Epidemiology and cause of severe acute respiratory syndrome (SARS) in Guangdong, People’s Republic of China, in February, 2003. Lancet.

[18] De Wit E., Van Doremalen N., Falzarano D., Munster V.J. (2016). SARS and MERS: Recent insights into emerging coronaviruses. Nat. Rev. Microbiol..

[19] Chan K., Zheng J., Mok Y., Li Y., Liu Y.N., Chu C., Ip M. (2003). SARS: Prognosis, outcome and sequelae. Respirology.

[20] World Health Organization (2020). Novel Coronavirus—Japan (ex-China). https://www.who.int/csr/don/17-january-2020-novel-coronavirus-japan-ex-china/en/.

[21] Wang C., Horby P.W., Hayden F.G., Gao G.F. (2020). A novel coronavirus outbreak of global health concern. Lancet.

[22] Guo Y.-R., Cao Q.-D., Hong Z.-S., Tan Y.-Y., Chen S.-D., Jin H.-J., Tan K.-S., Wang D.-Y., Yan Y. (2020). The origin, transmission and clinical therapies on coronavirus disease 2019 (COVID-19) outbreak—An update on the status. Mil. Med. Res..

[23] Chakraborty C., Sharma A.R., Bhattacharya M., Sharma G., Lee S.-S. (2020). The 2019 novel coronavirus disease (COVID-19) pandemic: A zoonotic prospective. Asian Pac. J. Trop. Med..

[24] Poon L.L., Peiris M. (2020). Emergence of a novel human coronavirus threatening human health. Nat. Med..

[25] Zhang T., Wu Q., Zhang Z. (2020). Probable pangolin origin of 2019-nCoV associated with outbreak of COVID-19. SSRN Electron. J..

[26] Rossi J.J., Rossi D. (2020). Oligonucleotides and the COVID-19 Pandemic: A Perspective. Nucleic Acid Ther..

[27] Zhong Y., Tan Y.W., Liu D.X. (2012). Recent progress in studies of arterivirus-and coronavirus-host interactions. Viruses.

[28] Li W., Shi Z., Yu M., Ren W., Smith C., Epstein J.H., Wang H., Crameri G., Hu Z., Zhang H. (2005). Bats Are Natural Reservoirs of SARS-Like Coronaviruses. Science.

[29] Gralinski L.E., Menachery V.D. (2020). Return of the Coronavirus: 2019-nCoV. Viruses.

[30] Lu R., Zhao X., Li J., Niu P., Yang B., Wu H., Wang W., Song H., Huang B., Zhu N. (2020). Genomic characterisation and epidemiology of 2019 novel coronavirus: Implications for virus origins and receptor binding. Lancet.

[31] Tang X., Wu C., Li X., Song Y., Yao X., Wu X., Duan Y., Zhang H., Wang Y., Qian Z. (2020). On the origin and continuing evolution of SARS-CoV-2. Natl. Sci. Rev..

[32] Dawood A.A. (2020). Mutated COVID-19, May Foretells Mankind in a Great Risk in the Future. New Microbes New Infect..

[33] Phan T. (2020). Genetic diversity and evolution of SARS-CoV-2. Infect. Genet. Evol..

[34] Wang Q., Qiu Y., Li J.-Y., Zhou Z.-J., Liao C.-H., Ge X.-Y. (2020). A unique protease cleavage site predicted in the spike protein of the novel pneumonia coronavirus (2019-nCoV) potentially related to viral transmissibility. Virol. Sin..

[35] Matyášek R., Kovařík A. (2020). Mutation Patterns of Human SARS-CoV-2 and Bat RaTG13 Coronavirus Genomes Are Strongly Biased Towards C> U Transitions, Indicating Rapid Evolution in Their Hosts. Genes.

[36] Lipsitch M., Swerdlow D.L., Finelli L. (2020). Defining the epidemiology of Covid-19—Studies needed. N. Engl. J. Med..

[37] Yuan H., Cao X., Ji X., Du F., Zhou X., He J., Xie Y., Zhu Y. (2020). A Current Emerging Respiratory Infection: Epidemiological and Clinical Characteristics, Diagnosis and Treatments of COVID-19. Diagnosis and Treatments of COVID-19. SSRN Electron. J..

[38] Huang C., Wang Y., Li X., Ren L., Zhao J., Hu Y., Zhang L., Fan G., Xu J., Gu X. (2020). Clinical features of patients infected with 2019 novel coronavirus in Wuhan, China. Lancet.

[39] Lu H. (2020). Drug treatment options for the 2019-new coronavirus (2019-nCoV). Biosci. Trends.

[40] Nishiura H., Jung S.-M., Linton N.M., Kinoshita R., Yang Y., Hayashi K., Kobayashi T., Yuan B., Akhmetzhanov A.R. (2020). The extent of transmission of novel coronavirus in Wuhan, China, 2020. J. Clin. Med..

[41] Rothan H.A., Byrareddy S.N. (2020). The epidemiology and pathogenesis of coronavirus disease (COVID-19) outbreak. J. Autoimmun..

[42] Epidemiology Working Group for NCIP Epidemic Response, Chinese Center for Disease Control and Prevention (2020). The epidemiological characteristics of an outbreak of 2019 novel coronavirus diseases (COVID-19) in China. Zhonghua Liu Xing Bing Xue Za Zhi.

[43] Xu B., Kraemer M.U., Group D.C. (2020). Open access epidemiological data from the COVID-19 outbreak. Lancet Infect. Dis..

[44] Nature. Coronavirus: The First Three Months as It Happened. https://www.nature.com/articles/d41586-020-00154-w.

[45] Read M.C. (2020). EID: High Contagiousness and Rapid Spread of Severe Acute Respiratory Syndrome Coronavirus 2. Emerg. Infect. Dis..

[46] Yang Y., Peng F., Wang R., Guan K., Jiang T., Xu G., Sun J., Chang C. (2020). The deadly coronaviruses: The 2003 SARS pandemic and the 2020 novel coronavirus epidemic in China. J. Autoimmun..

[47] Dowd J.B., Andriano L., Brazel D.M., Rotondi V., Block P., Ding X., Liu Y., Mills M.C. (2020). Demographic science aids in understanding the spread and fatality rates of COVID-19. Proc. Natl. Acad. Sci. USA.

[48] Wu Z., McGoogan J.M. (2020). Characteristics of and important lessons from the coronavirus disease 2019 (COVID-19) outbreak in China: Summary of a report of 72 314 cases from the Chinese Center for Disease Control and Prevention. JAMA.

[49] Peiris J., Guan Y., Yuen K. (2004). Severe acute respiratory syndrome. Nat. Med..

[50] Jahangir M.A. (2020). Coronavirus (COVID-19): History, Current Knowledge and Pipeline Medications. Int. J. Pharm. Pharmacol..

[51] He Y., Zhou Y., Liu S., Kou Z., Li W., Farzan M., Jiang S. (2004). Receptor-binding domain of SARS-CoV spike protein induces highly potent neutralizing antibodies: Implication for developing subunit vaccine. Biochem. Biophys. Res. Commun..

[52] Kuhn J., Li W., Choe H., Farzan M. (2004). Angiotensin-converting enzyme 2: A functional receptor for SARS coronavirus. Cell. Mol. Life Sci..

[53] Wu F., Zhao S., Yu B., Chen Y.-M., Wang W., Song Z.-G., Hu Y., Tao Z.-W., Tian J.-H., Pei Y.-Y. (2020). A new coronavirus associated with human respiratory disease in China. Nature.

[54] Simmons G., Reeves J.D., Rennekamp A.J., Amberg S.M., Piefer A.J., Bates P. (2004). Characterization of severe acute respiratory syndrome-associated coronavirus (SARS-CoV) spike glycoprotein-mediated viral entry. Proc. Natl. Acad. Sci. USA.

[55] Belouzard S., Chu V.C., Whittaker G.R. (2009). Activation of the SARS coronavirus spike protein via sequential proteolytic cleavage at two distinct sites. Proc. Natl. Acad. Sci. USA.

[56] Perlman S., Netland J. (2009). Coronaviruses post-SARS: Update on replication and pathogenesis. Nat. Rev. Genet..

[57] Guan W.-J., Ni Z.-Y., Hu Y., Liang W.-H., Ou C.-Q., He J.-X., Liu L., Shan H., Lei C.-L., Hui D.S. (2020). Clinical characteristics of coronavirus disease 2019 in China. N. Engl. J. Med..

[58] Mehta P., McAuley D.F., Brown M., Sanchez E., Tattersall R.S., Manson J.J. (2020). COVID-19: Consider cytokine storm syndromes and immunosuppression. Lancet.

[59] Siddiqi H.K., Mehra M.R. (2020). COVID-19 illness in native and immunosuppressed states: A clinical-therapeutic staging proposal. J. Hear. Lung Transplant..

[60] Tay M.Z., Poh C.M., Rénia L., MacAry P.A., Ng L.F. (2020). The trinity of COVID-19: Immunity, inflammation and intervention. Nat. Rev. Immunol..

[61] Zhang B., Zhou X., Qiu Y., Feng F., Feng J., Jia Y., Zhu H., Hu K., Liu J., Liu Z. (2020). Clinical characteristics of 82 death cases with COVID-19. medRxiv.

[62] Kupferschmidt K., Cohen J. (2020). Race to find COVID-19 treatments accelerates. Science.

[63] Hatami N., Ahi S., Sadeghinikoo A., Foroughian M., Javdani F., Kalani N., Fereydoni M., Keshavarz P. (2020). Worldwide ACE (I/D) Polymorphism May Affect COVID-19 Recovery Rate: An Ecological Meta-Regression.

[64] Backer J.A., Klinkenberg D., Wallinga J. (2020). Incubation period of 2019 novel coronavirus (2019-nCoV) infections among travellers from Wuhan, China, 20–28 January 2020. Eurosurveillance.

[65] Ng M.-Y., Lee E.Y., Yang J., Yang F., Li X., Wang H., Lui M.M.-S., Lo C.S.-Y., Leung B., Khong P.-L. (2020). Imaging profile of the COVID-19 infection: Radiologic findings and literature review. Radiol. Cardiothorac. Imaging.

[66] Wang Y., Wang Y., Chen Y., Qin Q. (2020). Unique epidemiological and clinical features of the emerging 2019 novel coronavirus pneumonia (COVID-19) implicate special control measures. J. Med. Virol..

[67] Pan X.-W., Xu H.Z.D., Zhou W., Wang L.-H., Cui X.-G. (2020). Identification of a potential mechanism of acute kidney injury during the COVID-19 outbreak: A study based on single-cell transcriptome analysis. Intensive Care Med..

[68] Zhang W., Zhao Y., Zhang F., Wang Q., Li T., Liu Z., Wang J., Qin Y., Zhang X., Yan X. (2020). The use of anti-inflammatory drugs in the treatment of people with severe coronavirus disease 2019 (COVID-19): The experience of clinical immunologists from China. Clin. Immunol..

[69] Kui L., Fang Y.-Y., Deng Y., Liu W., Wang M.-F., Ma J.-P., Xiao W., Wang Y.-N., Zhong M.-H., Li C.-H. (2020). Clinical characteristics of novel coronavirus cases in tertiary hospitals in Hubei Province. Chin. Med. J..

[70] Xu J., Zhao S., Teng T., Abdalla A.E., Zhu W., Xie L., Wang Y., Guo X. (2020). Systematic comparison of two animal-to-human transmitted human coronaviruses: SARS-CoV-2 and SARS-CoV. Viruses.

[71] Vaira L.A., Salzano G., Deiana G., De Riu G. (2020). Anosmia and ageusia: Common findings in COVID-19 patients. Laryngoscope.

[72] Kaye R., Chang C.D., Kazahaya K., Brereton J., Denneny J.C. (2020). COVID-19 anosmia reporting tool: Initial findings. Otolaryngol. Neck Surg..

[73] Klopfenstein T., Toko L., Royer P.-Y., Lepiller Q., Gendrin V., Zayet S. (2020). Features of anosmia in COVID-19. Médecine et Maladies Infectieuses.

[74] Manheim D., Denkenberger D. (2020). Review of Potential High-Leverage and Inexpensive Mitigations for Reducing Risk in Epidemics and Pandemics. J. Glob. Health Rep..

[75] World Health Organization (2004). WHO Guidelines for the Global Surveillance of Severe Acute Respiratory Syndrome (SARS): Updated Recommendations, October 2004.

[76] Zu Z.Y., Jiang M.D., Xu P.P., Chen W., Ni Q.Q., Lu G.M., Zhang L.J. (2020). Coronavirus disease 2019 (COVID-19): A perspective from China. Radiology.

[77] Azhar E.I., El-Kafrawy S.A., Farraj S.A., Hassan A.M., Al-Saeed M.S., Hashem A.M., Madani T.A. (2014). Evidence for camel-to-human transmission of MERS coronavirus. N. Engl. J. Med..

[78] World Health Organization (2020). Coronavirus Disease 2019: Situation Report, 51. https://www.who.int/docs/default-source/coronaviruse/situation-reports/20200311-sitrep-51-covid-19.pdf?sfvrsn=1ba62e57_10.

[79] Peeling R.W., Wedderburn C.J., Garcia P.J., Boeras D., Fongwen N., Nkengasong J., Sall A., Tanuri A., Heymann D.L. (2020). Serology testing in the COVID-19 pandemic response. Lancet Infect. Dis..

[80] Zhao J., Yuan Q., Wang H., Liu W., Liao X., Su Y., Wang X., Yuan J., Li T., Li J. (2020). Antibody responses to SARS-CoV-2 in patients of novel coronavirus disease 2019. Clin. Infect. Dis..

[81] Deng S.-Q., Peng H.-J. (2020). Characteristics of and public health responses to the coronavirus disease 2019 outbreak in China. J. Clin. Med..

[82] Yang W., Yan F. (2020). Patients with RT-PCR confirmed COVID-19 and normal chest CT. Radiology.

[83] Chan J.F.-W., Yip C.C.-Y., To K.K.-W., Tang T.H.-C., Wong S.C.-Y., Leung K.-H., Fung A.Y.-F., Ng A.C.-K., Zou Z., Tsoi H.-W. (2020). Improved molecular diagnosis of COVID-19 by the novel, highly sensitive and specific COVID-19-RdRp/Hel real-time reverse transcription-polymerase chain reaction assay validated in vitro and with clinical specimens. J. Clin. Microbiol..

[84] Mardani R., Vasmehjani A.A., Zali F., Gholami A., Nasab S.D.M., Kaghazian H., Kaviani M., Ahmadi N. (2020). Laboratory parameters in detection of COVID-19 patients with positive RT-PCR; a diagnostic accuracy study. Arch. Acad. Emerg. Med..

[85] Qu R., Ling Y., Zhang Y.H.Z., Wei L.Y., Chen X., Li X.M., Liu X.Y., Liu H.M., Guo Z., Ren H. (2020). Platelet-to-lymphocyte ratio is associated with prognosis in patients with coronavirus disease-19. J. Med. Virol..

[86] Yang X., Yu Y., Xu J., Shu H., Xia J., Liu H., Wu Y., Zhang L., Yu Z., Fang M. (2020). Clinical course and outcomes of critically ill patients with SARS-CoV-2 pneumonia in Wuhan, China: A single-centered, retrospective, observational study. Lancet Respir. Med..

[87] Zhang G., Hu C., Luo L., Fang F., Chen Y., Li J., Peng Z., Pan H. (2020). Clinical features and short-term outcomes of 221 patients with COVID-19 in Wuhan, China. J. Clin. Virol..

[88] Rodriguez-Morales A.J., Cardona-Ospina J.A., Gutiérrez-Ocampo E., Villamizar-Peña R., Holguin-Rivera Y., Escalera-Antezana J.P., Alvarado-Arnez L.E., Bonilla-Aldana D.K., Franco-Paredes C., Henao-Martinez A.F. (2020). Clinical, laboratory and imaging features of COVID-19: A systematic review and meta-analysis. Travel Med. Infect. Dis..

[89] Singhal T. (2020). A review of coronavirus disease-2019 (COVID-19). Indian J. Pediatr..

[90] Wang M., Cao R., Zhang L., Yang X., Liu J., Xu M., Shi Z., Hu Z., Zhong W., Xiao G. (2020). Remdesivir and chloroquine effectively inhibit the recently emerged novel coronavirus (2019-nCoV) in vitro. Cell Res..

[91] Park M., Thwaites R.S., Openshaw P.J. (2020). COVID-19: Lessons from SARS and MERS. Eur. J. Immunol..

[92] Yan Y., Shin W.I., Pang Y.X., Meng Y., Lai J., You C., Zhao H., Lester E., Wu T., Pang C.H. (2020). The First 75 Days of Novel Coronavirus (SARS-CoV-2) Outbreak: Recent Advances, Prevention, and Treatment. Int. J. Environ. Res. Public Health.

[93] Merad M., Martin J.C. (2020). Pathological inflammation in patients with COVID-19: A key role for monocytes and macrophages. Nat. Rev. Immunol..

[94] Ye Q., Wang B., Mao J. (2020). The pathogenesis and treatment of the Cytokine Storm’in COVID-19. J. Infect..

[95] Ye Q., Wang B., Mao J. (2020). Cytokine storm in COVID-19 and treatment. J. Infect..

[96] Serbina N.V., Pamer E.G. (2006). Monocyte emigration from bone marrow during bacterial infection requires signals mediated by chemokine receptor CCR2. Nat. Immunol..

[97] Stebbing J., Phelan A., Griffin I., Tucker C., Oechsle O., Smith D., Richardson P. (2020). COVID-19: Combining antiviral and anti-inflammatory treatments. Lancet Infect. Dis..

[98] Feldmann M., Maini R.N., Woody J.N., Holgate S.T., Winter G., Rowland M., Richards D., Hussell T. (2020). Trials of anti-tumour necrosis factor therapy for COVID-19 are urgently needed. Lancet.

[99] Wu D., Yang X.O. (2020). TH17 responses in cytokine storm of COVID-19: An emerging target of JAK2 inhibitor Fedratinib. J. Microbiol. Immunol. Infect..

[100] De Wit E., Feldmann F., Cronin J., Jordan R., Okumura A., Thomas T., Scott D., Cihlar T., Feldmann H. (2020). Prophylactic and therapeutic remdesivir (GS-5734) treatment in the rhesus macaque model of MERS-CoV infection. Proc. Natl. Acad. Sci. USA.

[101] Parang K., El-Sayed N.S., Kazeminy A.J., Tiwari R.K. (2020). Comparative Antiviral Activity of Remdesivir and Anti-HIV Nucleoside Analogs against Human Coronavirus 229E (HCoV-229E). Molecules.

[102] Paules C.I., Marston H.D., Fauci A.S. (2020). Coronavirus infections—More than just the common cold. JAMA.

[103] Memoli M.J., Han A., Walters K.-A., Czajkowski L., Reed S., Athota R., Angela Rosas L., Cervantes-Medina A., Park J.-K., Morens D.M. (2019). Influenza A reinfection in sequential human challenge: Implications for protective immunity and “Universal” vaccine development. Clin. Infect. Dis..

[104] McMullan L.K. (2020). Clinical trials in an Ebola outbreak seek to find an evidence-based cure. EBioMedicine.

[105] Grein J., Ohmagari N., Shin D., Diaz G., Asperges E., Castagna A., Feldt T., Green G., Green M.L., Lescure F.-X. (2020). Compassionate Use of Remdesivir for Patients with Severe Covid-19. N. Engl. J. Med..

[106] Food and Drug Administration (FDA) (2020). Coronavirus (COVID-19) Update: FDA Issues Emergency Use Authorization for Potential COVID-19 Treatment. https://www.fda.gov/news-events/press-announcements/coronavirus-covid-19-update-fda-issues-emergency-use-authorization-potential-covid-19-treatment.

[107] Dong L., Hu S., Gao J. (2020). Discovering drugs to treat coronavirus disease 2019 (COVID-19). Drug Discov. Ther..

[108] Cao B., Wang Y., Wen D., Liu W., Wang J., Fan G., Ruan L., Song B., Cai Y., Wei M. (2020). A trial of lopinavir–ritonavir in adults hospitalized with severe Covid-19. N. Engl. J. Med..

[109] Peng F., Tu L., Yang Y., Hu P., Wang R., Hu Q., Cao F., Jiang T., Sun J., Xu G. (2020). Management and Treatment of COVID-19: The Chinese Experience. Can. J. Cardiol..

[110] Tobaiqy M., Qashqary M., Al-Dahery S., Mujallad A., Hershan A.A., Kamal M.A., Helmi N. (2020). Therapeutic Management of COVID-19 Patients: A systematic review. Infect. Prev. Pract..

[111] Theoharides T., Conti P. (2020). Dexamethasone for COVID-19? Not so fast. J. Biol. Regul. Homeost. Agents.

[112] Johnson R.M., Vinetz J.M. (2020). Dexamethasone in the management of covid-19. Br. Med. J..

[113] Recovery Collaborative Group (2020). Dexamethasone in Hospitalized Patients with Covid-19—Preliminary Report. N. Engl. J. Med..

[114] Whitty C. (2020). Dexamethasone in the Treatment of COVID-19: Implementation and Management of Supply for Treatment in Hospitals.

[115] Bethesda M. COVID-19 Treatment Guidelines. https://www.covid19treatmentguidelines.nih.gov/dexamethasone/.

[116] Gautret P., Lagier J.-C., Parola P., Meddeb L., Mailhe M., Doudier B., Courjon J., Giordanengo V., Vieira V.E., Dupont H.T. (2020). Hydroxychloroquine and azithromycin as a treatment of COVID-19: Results of an open-label non-randomized clinical trial. Int. J. Antimicrob. Agents.

[117] Yao X., Ye F., Zhang M., Cui C., Huang B., Niu P., Liu X., Zhao L., Dong E., Song C. (2020). In vitro antiviral activity and projection of optimized dosing design of hydroxychloroquine for the treatment of severe acute respiratory syndrome coronavirus 2 (SARS-CoV-2). Clin. Infect. Dis..

[118] Cavalcanti A.B., Zampieri F.G., Rosa R.G., Azevedo L.C., Veiga V.C., Avezum A., Damiani L.P., Marcadenti A., Kawano-Dourado L., Lisboa T. (2020). Hydroxychloroquine with or without Azithromycin in Mild-to-Moderate Covid-19. N. Engl. J. Med..

[119] Klok F., Kruip M., Van der Meer N., Arbous M., Gommers D., Kant K., Kaptein F., Van Paassen J., Stals M., Huisman M. (2020). Incidence of thrombotic complications in critically ill ICU patients with COVID-19. Thromb. Res..

[120] Cattaneo M., Bertinato E.M., Birocchi S., Brizio C., Malavolta D., Manzoni M., Muscarella G., Orlandi M. (2020). Pulmonary embolism or pulmonary thrombosis in COVID-19? Is the recommendation to use high-dose heparin for thromboprophylaxis justified?. Thromb. Haemost..

[121] Barrett C.D., Moore H.B., Yaffe M.B., Moore E.E. (2020). ISTH interim guidance on recognition and management of coagulopathy in COVID-19: A Comment. J. Thromb. Haemost..

[122] Shetty R., Ghosh A., Honavar S.G., Khamar P., Sethu S. (2020). Therapeutic opportunities to manage COVID-19/SARS-CoV-2 infection: Present and future. Indian J. Ophthalmol..

[123] Hoffmann M., Schroeder S., Kleine-Weber H., Müller M.A., Drosten C., Pöhlmann S. (2020). Nafamostat mesylate blocks activation of SARS-CoV-2: New treatment option for COVID-19. Antimicrob. Agents Chemother..

[124] Molina J.M., Delaugerre C., Goff J., Mela-Lima B., Ponscarme D., Goldwirt L., De Castro N. (2020). No evidence of rapid antiviral clearance or clinical benefit with the combination of hydroxychloroquine and azithromycin in patients with severe COVID-19 infection. Med. Mal. Infect..

[125] Jin Y., Yang H., Ji W., Wu W., Chen S., Zhang W., Duan G. (2020). Virology, epidemiology, pathogenesis, and control of COVID-19. Viruses.

[126] Roback J.D., Guarner J. (2020). Convalescent plasma to treat COVID-19: Possibilities and challenges. JAMA.

[127] Lythgoe M.P., Middleton P. (2020). Ongoing clinical trials for the management of the COVID-19 pandemic. Trends Pharmacol. Sci..

[128] Scholz M., Derwand R. (2020). Does Zinc Supplementation Enhance the Clinical Efficacy of Chloroquine/Hydroxychloroquine to Win Todays Battle Against COVID-19?. Med. Hypotheses.

[129] Santacroce L., Charitos I., Del Prete R. (2020). COVID-19 in Italy: An overview from the first case to date. Electron. J. Gen. Med..

[130] Connors J.M., Levy J.H. (2020). Thromboinflammation and the hypercoagulability of COVID-19. J. Thromb. Haemost..

[131] Gupta R., Ghosh A., Singh A.K., Misra A. (2020). Clinical considerations for patients with diabetes in times of COVID-19 epidemic. Diabetes Metab. Syndr..

[132] Grant W.B., Lahore H., McDonnell S.L., Baggerly C.A., French C.B., Aliano J.L., Bhattoa H.P. (2020). Evidence that vitamin D supplementation could reduce risk of influenza and COVID-19 infections and deaths. Nutrients.

[133] Le T.T., Andreadakis Z., Kumar A., Román R.G., Tollefsen S., Saville M., Mayhew S. (2020). The COVID-19 vaccine development landscape. Nat. Rev. Drug Discov..

[134] Ota M. (2020). Will we see protection or reinfection in COVID-19?. Nat. Rev. Immunol..

[135] World Health Organization (2020). Rational Use of Personal Protective Equipment for Coronavirus Disease (COVID-19): Interim Guidance, 27 February 2020.

[136] Adams J.G., Walls R.M. (2020). Supporting the health care workforce during the COVID-19 global epidemic. JAMA.

[137] Zhang X., Shao F., Lan X. (2020). Suggestions for safety and protection control in Department of Nuclear Medicine during the outbreak of COVID-19. Eur. J. Nucl. Med. Mol. Imaging.

[138] Hamid S., Mir M.Y., Rohela G.K. (2020). Noval coronavirus disease (COVID-19): A pandemic (Epidemiology, Pathogenesis and potential therapeutics). New Microbes New Infect..

